# Evaluation of an artificial intelligence model based on multiparametric transrectal ultrasound for localizing clinically significant prostate cancer by simulation of targeted biopsies

**DOI:** 10.1007/s00330-025-12114-x

**Published:** 2025-11-06

**Authors:** Daniel L. van den Kroonenberg, Florian Delberghe, Auke Jager, Arnoud W. Postema, Katelijne C. C. de Bie, Johannes B. Reitsma, Marije Zwart, Hessel Wijkstra, Anna Garrido-Utrilla, Joost de Baaij, Jean-Paul A. van Basten, Henk G. van der Poel, Harrie P. Beerlage, Massimo Mischi, Jorg R. Oddens

**Affiliations:** 1 Department of Urology, Amsterdam UMC, Amsterdam, The Netherlands; 2 Cancer Centre Amsterdam, Amsterdam, The Netherlands; 3 Lab of Biomedical Diagnostics, Department of Electrical Engineering, Eindhoven University of Technology, Eindhoven, The Netherlands; 4 Department of Urology, Leiden University Medical Center, Leiden, The Netherlands; 5 Julius Center for Health Sciences and Primary Care, UMC Utrecht, Utrecht University, Utrecht, The Netherlands; 6 Angiogenesis Analytics, Den Bosch, The Netherlands; 7 Department of Urology, Canisius Wilhelmina Hospital, Nijmegen, The Netherlands; 8 Prosper Collaborative Prostate Cancer Clinics, Nijmegen-Eindhoven, The Netherlands; 9 Department of Urology, Netherlands Cancer Institute - Antoni van Leeuwenhoek Hospital, Amsterdam, The Netherlands

**Keywords:** Prostate cancer, Multiparametric ultrasound, Contrast enhanced ultrasound, Artificial intelligence, Full mount prostatectomy histopathology

## Abstract

**Introduction and objectives:**

An AI model that performs well during training does not guarantee similar performance in clinical practice and should be carefully evaluated before implementation. We aimed to evaluate a voxel-level trained AI model (AUROC 0.87), which utilizes a three-dimensional multiparametric transrectal prostate ultrasound (3D mpUS) to identify clinically significant prostate cancer (csPCa).

**Materials and methods:**

We included patients with csPCa (Grade Group ≥ 2 and scheduled for radical prostatectomy (RP)) and without csPCa (PI-RADS ≤ 2 and/or negative systematic biopsies). Histopathology of RP specimens provided the csPCa reference standard. 3D mpUS consisted of grayscale, contrast-enhanced ultrasound, and shear-wave elastography using automated acquisition. We assessed patient-level diagnostic accuracy by comparing the results of simulated targeted biopsies based on the AI model with the reference standard in internal and external evaluation. Patients without csPCa and RP reference standard were used to determine specificity.

**Results:**

Based on internal evaluation of 250 patients, a sensitivity of 0.82 (CI 0.75 to 0.87) and specificity of 0.43 (CI 0.32 to 0.55) was reached for ISUP ≥ 2. For ISUP ≥ 3, this was 0.90 (CI 0.83–0.95) and 0.39 (CI 0.31–0.47). In the external evaluation of 77 patients, the sensitivity for ISUP ≥ 2 was 0.81 (CI 0.65–0.90), with a specificity of 0.42 (CI 0.28–0.57). For ISUP ≥ 3, this was 0.96 (CI 0.78–0.99) and 0.42 (CI 0.30–0.55).

**Conclusions:**

The AI model based on 3D mpUS showed consistent patient-level performance for csPCa detection in internal and external evaluation, comparable to voxel-level analysis. These suggest strong generalizability and support prospective clinical trials.

**Trial registration:**

NCT04605276.

**Key Points:**

***Question*** *Does the diagnostic performance of a 3D multiparametric ultrasound-based AI model translate from voxel-level training to patient-level biopsy simulation?*

***Findings*** *Simulated biopsy performance aligned with voxel-level results, showing robust csPCa detection and supporting the model’s generalizability across independent datasets.*

***Clinical relevance*** *The AI model’s consistent biopsy simulation performance confirms its readiness for clinical evaluation and suggests diagnostic value in MRI-constrained settings.*

**Graphical Abstract:**

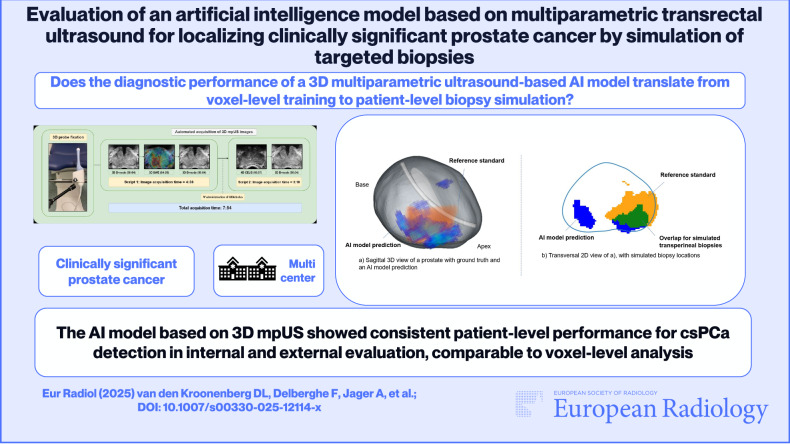

## Introduction

The current diagnostic approach in primary prostate cancer (PCa) relies on MRI, followed by targeted prostate biopsies (TBx) when indicated. The introduction of MRI has demonstrated improved patient selection prior to biopsy, resulting in decreased detection of insignificant PCa (isPCa) and improved detection of clinically significant PCa (csPCa) [1–3]. However, MRI has several limitations, related to diagnostic accuracy, inter-observer variability, availability, and cost [4]. Recent advances in ultrasound (US) imaging, such as multiparametric ultrasound (mpUS) and micro-US, have demonstrated promising diagnostic performance and may provide a valuable alternative [5–9]; however, the lack of standardized image acquisition and interpretation limits clinical implementation [10–12]. This challenge can be overcome by automated interpretation, provided by a computer-aided diagnosis (CAD) system powered by artificial intelligence (AI) models [13].

Recently, our AI model based on mpUS for csPCa has demonstrated promising diagnostic performance (AUROC of 0.87) when evaluated at voxel (0.75^3 ^mm³) level [14, 15]. However, an AI model that performs well during voxel-level evaluation does not necessarily translate to equivalent accuracy based on clinical TBx procedure due to, e.g., procedural variability, sampling errors, or spatial discrepancies between the model and the actual tumor [16, 17]. As the model is ultimately intended to guide prostate biopsy procedures [18], we aim to validate the performance at the patient level as well. The model processes mpUS images and generates a heatmap highlighting areas of interest for targeting biopsies. The objective of this study was to perform a patient-level evaluation in an internal and external cohort of the mpUS AI model for csPCa detection by simulation of TBx performance and a comparison to voxel-level performance. Simulating biopsies offers a controlled means of estimating the AI model’s clinical performance and determining whether the model is suitable for prospective clinical evaluation.

## Materials and methods

### Design and study population

**Fig. 1:**
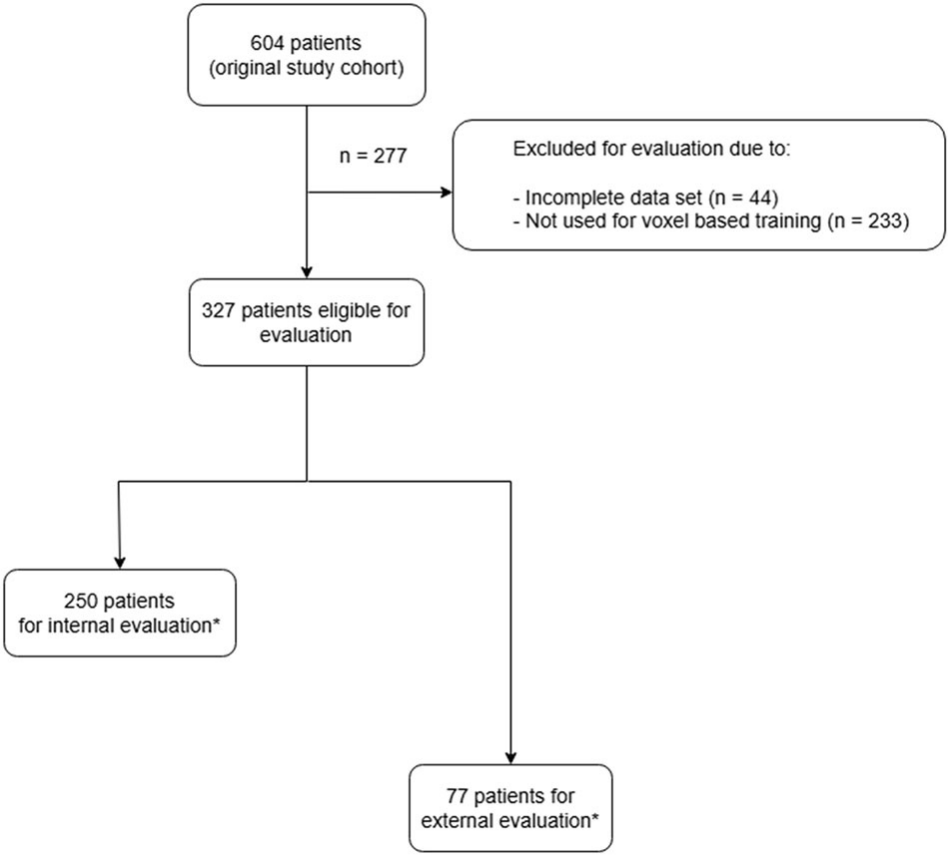
Inclusion and exclusion flowchart for internal and external validation. * Group selection was based on the time of inclusion. Patients enrolled before the AI model training was completed were used in the training process and thus included in the internal evaluation cohort. After training reached performance saturation at the voxel level, all subsequently included patients—who had no influence on model development—were assigned to the external evaluation cohort

This is a retrospective study to validate the patient-level diagnostic performance of an AI model based on 3D mpUS for csPCa detection (NCT04605276). Evaluation was conducted in two phases: internal evaluation using cross-validation within the training cohort, and external evaluation on patients not involved in model development (Fig. 1). Selection of these cohorts was based on the point at which training was halted due to performance saturation. However, the training process itself was not part of this study.

The study population included men who either (1) had biopsy-proven csPCa and were scheduled for radical prostatectomy (RP) or (2) had negative MRI (PI-RADS ≤ 2) or negative prostate biopsies. Detailed descriptions of the training, inclusion/exclusion criteria, acquisition, and reference standard are available in the protocol and Supplementary Material [10]. All patients provided written informed consent for the use of their data in the development phase and for inclusion in this retrospective evaluation study.

### Simulating biopsy procedure

**Fig. 2:**
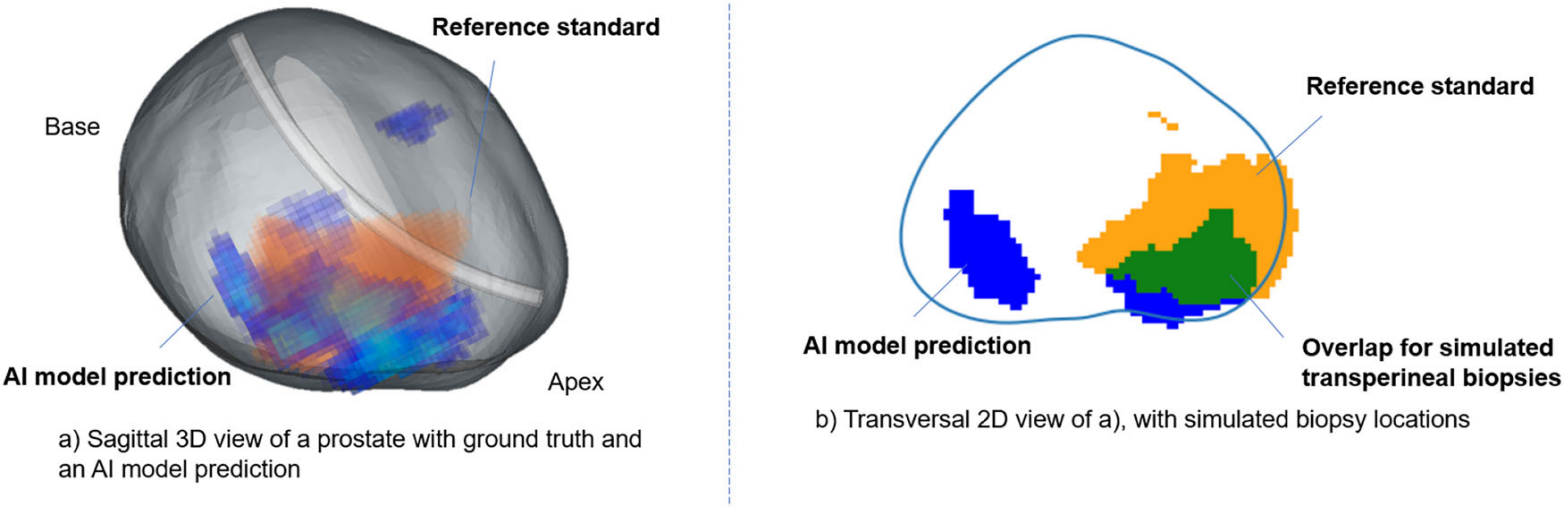
Simulated targeted biopsy procedure. 3D, three-dimensional; 2D, two-dimensional; AI, artificial intelligence

We simulated TBx based on the model’s heatmap to estimate the model’s potential performance in a real-world clinical setting (Fig. 2). First, we identified automatically predicted lesions ≥ 0.07 cc from the heatmap, and selected the two largest ones for simulated biopsy. To simplify the simulation, we assumed that biopsy needles are inserted in a straight path through the prostate, like transperineal biopsies performed using a brachytherapy grid. This allowed us to reduce the analysis to two dimensions by projecting both the predicted and actual lesions onto a single 2D biopsy plane. This plane shows a transverse view of the prostate and is perpendicular to the needle direction. Finally, we calculated the chance of a positive biopsy by calculating the overlap between the predicted lesions and the reference standard lesions derived from histology of the full prostate on the 2D plane. A biopsy was considered a true positive if the probability exceeded 0.5. Any lesion detected by the AI model in patients without csPCa was classified as false positive.

### Accuracy measure outcomes and statistical analysis

The primary outcome was the simulated diagnostic performance of the AI model, determined by internal (cross) and external evaluation. Diagnostic performance on the patient level was evaluated by calculating sensitivity, specificity, positive predictive value (PPV), and negative predictive value (NPV). Descriptive analyses were performed using STATA (version 16). Non-normally distributed data are presented as median and interquartile range (IQR).

## Results

A total of 327 patients were included to validate the AI model, comprising 250 in the internal evaluation cohort for cross-validation and 77 in the external evaluation cohort (Table 1). In the internal cohort, PI-RADS ≤ 2 was found in 28% of patients, PI-RADS 3 in 5.2%, and PI-RADS 4–5 in 67%. In the external cohort, 53% had PI-RADS ≤ 2, 2.6% had PI-RADS 3, and 44% had PI-RADS 4–5.

**Table 1:** Clinicopathological characteristics (*n* = 327)

	Internal evaluation cohort (*n* = 250)	External evaluation cohort (*n* = 77)
Age; median (IQR)	69 (64–73)	69 (66–73)
Last serum PSA (ng/mL); median (IQR)	8.0 (6.0–12)	8.1 (5.4–10)
Digital rectal exam (DRE); *N* (%)		
Benign	151 (60)	50 (65)
cT2	91 (37)	25 (31)
cT3/4	4 (2)	2 (2.6)
Missing	4 (2)	2 (2.6)
MRI prostate volume (mL); median (IQR)	47 (36–74)	52 (41–82)
PSA density (median (IQR))	0.16 (0.11–0.25)	0.13 (0.09–0.20)
MRI PI-RADS score; *N* (%)		
PI-RADS ≤ 2	69 (28)	41 (53)
PI-RADS 3	12 (5.2)	2 (2.6)
PI-RADS 4	76 (30)	11 (14)
PI-RADS 5	92 (37)	23 (30)
No MRI	1	
Radiological tumor stage; *N* (%)		
mT2	104 (42)	20 (26)
mT3a	48 (19)	13 (17)
mT3b	10 (4)	2 (3)
mT4	0	0
Not reported	88 (35)	41 (53)
ISUP grade group from prostatectomy; *N* (%)		
1	2 (1)	1 (1)
2	84 (34)	14 (18)
3	71 (28)	13 (17)
4	1	0
5	21 (8)	8 (10)

*PSA* prostate-specific antigen, *DRE* digital rectal exam, *3D mpUS* three-dimensional multiparametric ultrasound, *ISUP* International Society of Urological Pathology

### Internal cohort

Internal evaluation was performed on 250 patients, of which 177 (71%) patients had ISUP ≥ 2 at RP, and 73 patients (30%) did not undergo RP. The simulated biopsies based on the AI algorithm based on mpUS demonstrated a sensitivity of 0.82 (95% CI 0.75 to 0.87) and a specificity of 0.43 (CI 0.32–0.55) for ISUP ≥ 2 (*n* = 175) (Table 2).

**Table 2:** Performance comparison in sensitivity (a); Performance comparison in specificity (b); Predictive values (c)

a		
	Sensitivity (internal cohort)	Sensitivity (external cohort)
ISUP ≥ 2	0.82 (CI 0.75–0.87) (*n*^#^ = 177)	0.81 (CI 0.65–0.90) (*n* = 35)
ISUP ≥ 3	0.90 (CI 0.83–0.95) (*n* = 93)	0.96 (CI 0.78–0.99) (*n* = 21)

b		
	Specificity (internal cohort)	Specificity (external cohort)
ISUP ≥ 2	0.43 (CI 0.32–0.55) (*n* = 73)	0.42 (CI 0.28–0.57) (*n* = 42)
ISUP ≥ 3	0.39 (CI 0.31–0.47) (*n* = 157)	0.42 (CI 0.30–0.55) (*n* = 56)

c				
	PPV (internal cohort)	PPV (external cohort)	NPV (internal cohort)	NPV (external cohort)
ISUP ≥ 2 (*n* = 177)	0.76 (CI 0.72–0.80)	0.55 (CI 0.42–0.67)	0.52 (CI 0.42–0.62)	0.71 (CI 0.51–0.85)
ISUP ≥ 3 (*n* = 93)	0.55 (CI 0.51–0.58)	0.40 (CI 0.28–0.53)	0.83 (CI 0.72–0.90)	0.96 (CI 0.80–0.99)

*ISUP* International Society of Urological Pathology, *PPV* positive predictive value, *NPV* negative predictive value

^#^ The total number of patients appropriate for the specified analysis

The PPV and NPV were 0.76 (CI 0.72–0.80) and 0.52 (CI 0.42–0.62), respectively. For ISUP ≥ 3 (*n* = 93), the sensitivity was 0.90 (CI 0.83–0.95) and the specificity was 0.39 (CI 0.31–0.47). The corresponding positive PPV and NPV were 0.55 (CI 0.51–0.58) and 0.83 (CI 0.72–0.90), respectively.

### External cohort

External evaluation was performed on 77 patients, of which 35 (45%) had ISUP ≥ 2 at RP, and 42 patients (55%) did not undergo RP. In this external evaluation, the AI algorithm had a sensitivity of 0.81 (CI 0.65–0.90) and a specificity of 0.42 (CI 0.28–0.57) for the detection of ISUP ≥ 2 (Table 2). The PPV and NPV were 0.55 (CI 0.42–0.67) and 0.71 (CI 0.51–0.85), respectively. For ISUP ≥ 3 (*n* = 22), sensitivity was 0.96 (CI 0.78–0.99) and 0.42 (CI 0.30–0.55). The PPV and NPV were 0.40 (CI 0.28–0.53) and 0.96 (CI 0.80–0.99), respectively.

## Discussion

The aim of the study was to estimate the clinical diagnostic performance of a mpUS AI model for detecting csPCa. To this end, we performed simulated targeted biopsies guided by model-generated heatmaps indicating suspicious lesions. The model demonstrated consistent simulated diagnostic performance for detecting csPCa during internal and external evaluation settings.

We found a sensitivity of 0.82 (CI 0.75–0.87) for detecting ISUP ≥ 2 in the internal evaluation cohort, and this was comparable to the voxel-based results (0.79). The specificity was lower compared to the voxel-based results (0.78) at 0.43 (CI 0.32–0.55). In voxel analysis, false-positive voxels are diluted across millions of voxels, minimizing their impact. In contrast, in biopsy simulation, a single false-positive AI prediction misclassifies an entire patient. This highlights the importance of this clinically relevant way of evaluation, emphasizing that the performance of a voxel-based model cannot be assumed to directly translate into equivalent performance in clinical practice. In intermediate to high-risk cases (ISUP ≥ 3), almost all patients (90%) were detected by the model. However, the detection rate for ISUP 2 was lower, identifying 70% (59/85) of cases. This might be explained by the relatively low volume of our ISUP 2 tumors, and because the AI model’s features are less discriminative in these relatively low-grade tumors. Furthermore, current discussions are ongoing about what the long-term impact on survival is of missing ISUP 2 tumors, as these tumors could, depending on PSA and clinical stage, often be classified as favorable to intermediate risk and possibly eligible for conservative management [19]. As for negative patients, both internal and external evaluation showed a relatively low specificity, indicating a high rate of benign or isPCa findings, and therefore unnecessary biopsies. This specificity is comparable to that of MRI, as described in the meta-analysis by Drost et al (specificity of 0.37) [20]. Nevertheless, the specificity of our model requires further improvement to enhance its clinical utility, irrespective of this similarity with MRI.

The external evaluation demonstrated robust results across all ISUP grade groups, closely aligning with the outcomes observed in the internal evaluation cohort. This consistency highlights the model’s strong generalizability for both specificity and sensitivity. Interestingly, sensitivity even slightly improved in the external evaluation. This improvement could be explained by the fact that the external patient cohort contains a higher proportion of higher-grade tumors, which are generally easier to detect. Nevertheless, the number of patients in this external evaluation is low, and results should be carefully interpreted. PPV and NPV were satisfactory in both internal and external evaluation. However, it is important to recognize that PPV and NPV are prevalence-dependent metrics. Given that our cohort was designed with an artificially high prevalence of PCa for training purposes, these results should be interpreted with caution.

An imaging pathway using mpUS with an AI model could potentially serve as an alternative or add-on to MRI in diagnosing csPCa, reducing costs and improving clinical workflow. A previous study that prospectively compared PCa detection rates based on contrast-enhanced US alone to MRI already showed a comparable sensitivity, albeit with lower specificity [8]. Furthermore, their study used no automated acquisition, only 2D images were taken, and no AI model was used in the analysis of the images. All these limitations have now been addressed through automated 3D acquisition and AI-driven interpretation, which improves both the generalizability and clinical applicability of the mpUS pathway. The current study does not allow direct comparison with MRI, as most patients received their primary initial diagnosis based on MRI and MRI-targeted biopsies, introducing a bias in favor of MRI. Currently ongoing head-to-head trials will directly assess the performance of the mpUS pathway compared to MRI in order to evaluate its value in a new diagnostic pathway [21]. In addition, future studies should incorporate a formal health-economic evaluation to compare the costs of the mpUS pathway with those of mpMRI.

This study has limitations to consider. By design, the dataset is not an accurate representation of the pre-biopsy population, since it holds a csPCa prevalence of 70%. This increased prevalence impacts all diagnostic performance metrics. This limitation, however, does not undermine the overall robustness of this algorithm in an external patient population. While the AI model’s diagnostic performance is satisfactorily high, this remains based on a simulation and may not fully represent its performance in clinical practice. In real-world practice, additional sources of error, such as targeting inaccuracies and image registration discrepancies, can negatively impact biopsy accuracy. Also, we did not perform a statistical comparison between the simulation and voxel-based results because the methods used to assess performance differ substantially. However, as the cohorts are identical, we can do an accurate estimation of both performances. We observed an imbalance between the internal and external cohorts, most notably in the distribution of PI-RADS categories. These differences reflect the result of the training phase, which was concluded once model performance saturated, rather than through deliberate selection. Such imbalances may have influenced diagnostic performance and should be considered when interpreting the results. It is important to acknowledge that patients were excluded from the original dataset for several reasons. The relatively high exclusion rate can largely be attributed to the stringent training requirements for image quality, following the principle of ‘garbage in, garbage out’ [22]. Moreover, evaluation requires complete datasets, whereas training can be conducted on different parts of mpUS images.

Furthermore, previous studies suggest that RP pathology may not always be concordant with biopsy pathology, which could impact the model’s performance when used in clinical practice [23]. Nevertheless, the robust performance in this study warrants clinical and prospective evaluation of the model. Acknowledging that our results could be improved, developments to further refine diagnostic performance are still ongoing to fully leverage the potential of AI in medical imaging (NCT06935487).

## Conclusion

The simulated targeted biopsy procedure based on the mpUS AI model shows robust results for detecting csPCa. They are comparable to the voxel-based results and indicate that performance is translatable to clinical practice. Prospective clinical evaluation is necessary to further investigate the diagnostic accuracy and compare it to MRI.

## Supplementary information

ELECTRONIC SUPPLEMENTARY MATERIAL

## Abbreviations

AUROC: Area under the receiver operating curve
csPCa: Clinically significant prostate cancer
CAD: Computer-aided diagnosis
isPCa: Insignificant PCa
ISUP: International Society of Urological Pathology
IQR: Interquartile range
mpUS: Multiparametric ultrasound
NPV: Negative predictive value
PPV: Positive predictive value
PCa: Prostate cancer
RP: Radical prostatectomy
TBx: Targeted prostate biopsies
3D mpUS: Three-dimensional multiparametric transrectal prostate ultrasound
