# Supplementary material for: Evaluation of an artificial intelligence model based on multiparametric transrectal ultrasound for localizing clinically significant prostate cancer by simulation of targeted biopsies
Source: Eur Radiol. 2025 Nov 6;36(4):3043–9. doi: 10.1007/s00330-025-12114-x (PMC13035562; doi:10.1007/s00330-025-12114-x)
Supplement: Supplementary file 1 — ELECTRONIC SUPPLEMENTARY MATERIAL [file 330_2025_12114_MOESM1_ESM.docx]

**Supplementary Material**

*Multiparametric ultrasound based AI model*

The 3D mpUS image sequence consisted out of 3D SWE, 3D B-mode, and 4D CEUS, acquired using a LOGIQ E10 with a RIC5-9 probe. The AI model was developed using 7-fold cross-validation, repeated five times, and the average predictions across these repetitions were used for both internal and external evaluation. The AI model generates a prediction heatmap as an overlay on the 3D mpUS images. Additional information on the development and voxel performance of the AI model is reported in a separate study (1). Prostates mpUS images were automatically segmented by an AI model, developed using the same patients (2).

*Reference standard*

For this study the reference standard was derived in separate ways for prostates that were considered to contain csPCa and those that were not. The non-csPCa reference standard consisted of patients with PI-RADS ≤ 2 on MRI (no biopsies) or negative systematic biopsies and TBx (regardless of MRI). For the csPCa reference standard, RP specimens from patients with biopsy proven csPCa were used. Patients with biopsy-proven csPCa scheduled for RP were selected and scanned preoperatively in the outpatient clinic. The RP specimens were subsequently processed according to a study-specific protocol, digitally reconstructed and registered to the 3D mpUS images (3, 4). Pathology annotations were performed using a web-based tool (Slidescore, Amsterdam, the Netherlands) according to a validated study specific protocol by dedicated uro-pathologists (3). Each lesion was individually classified according to the International Society of Urological Pathology (ISUP) guidelines; csPCa was defined as ISUP Grade Group (GG) ≥ 2 with a volume of ≥ 0.3 ml (5).

Image acquisition sequence with time (min:sec) per ultrasound modality*


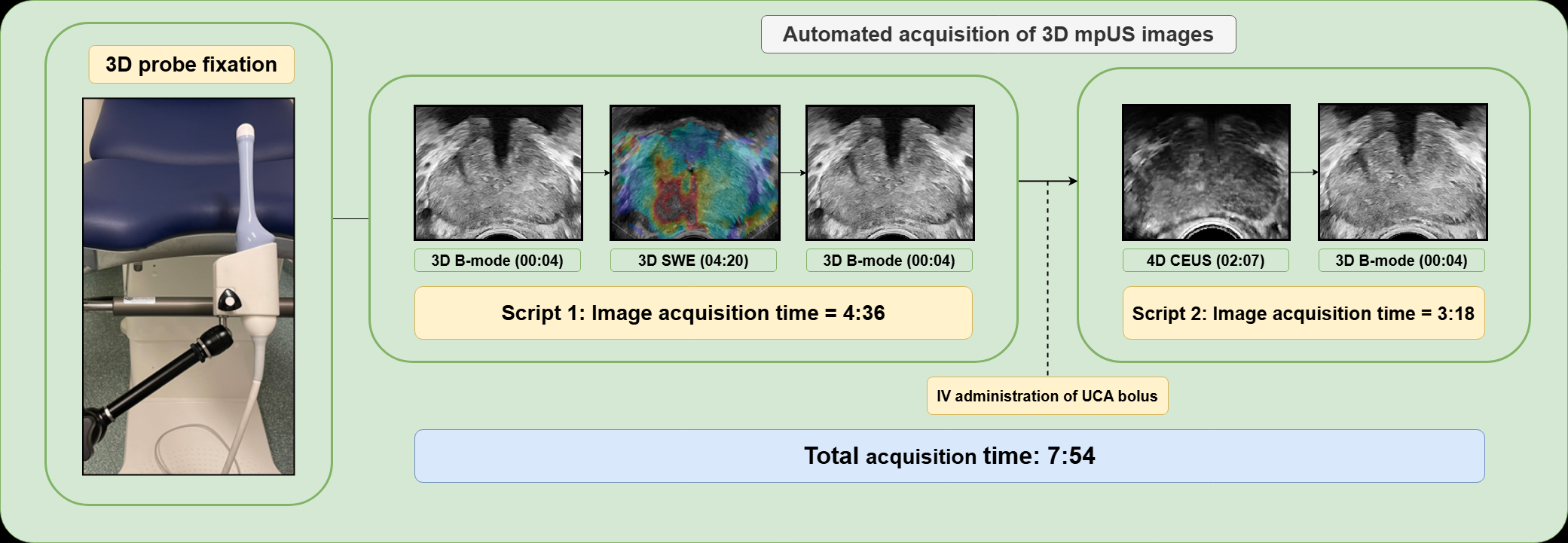


B-Mode: Brightness-mode; SWE: Shear wave elastography, IV: Intravenous; UCA: ultrasound contrast agent; CEUS= Contrast Enhanced Ultrasound

* van den Kroonenberg, D. L., Went, J., Jager, A., Garrido-Utrilla, A., Trappenburg, J. C. A., Postema, A. W., … Oddens, Jorg. R. (2025). Developing a training for 3D transrectal multiparametric ultrasound of the prostate: a human factors engineering approach. *Expert Review of Medical Devices*, *22*(4), 361–367. <https://doi.org/10.1080/17434440.2025.2473632>

*Table 1a: Settings for 4D Contrast Enhanced Ultrasound*

| **Setting** | **Value** |
| --- | --- |
| Axial voxel size (mm) | 0.14 – 0.17 |
| Dynamic range (db) | 42 |
| Gain (db) | 55 |
| Power level (%) | 10 |
| Transducer frequency (kHz) | 3500 |
| Mechanical index | 0.10 |
| Radius start (mm) | 12.389 – 15.2 |
| Radius mean step size (mm) | 0.14 – 0.17 |
| Radius min step size (mm) | 0.14 – 0.17 |
| Radius max step size (mm) | 0.14 – 0.17 |
| Radius jitter (mm) | 0.0 |
| Azimuth range (deg) | 110 – 150 |
| Azimuth mean step size (mm) | 0.79 – 0.81 |
| Azimuth min step size (mm) | 0.79 – 0.81 |
| Azimuth max step size (mm) | 0.79 – 0.81 |
| Azimuth jitter (mm) | 0.0 |
| Elevation range (deg) | 118 – 120 |
| Elevation mean step size (mm) | 2.30 – 2.372 |
| Elevation min step size (mm) | 2.30 – 2.372 |
| Elevation max step size (mm) | 2.30 – 2.372 |
| Elevation jitter (mm) | 0.0 |

Table 1b: Settings for B-Mode

| **Setting** | **Value** |
| --- | --- |
| Axial voxel size (mm) | 0.05 – 0.14 |
| Dynamic range (db) | 69 |
| Gain (db) | 55 |
| Power level (%) | 100 |
| Transducer frequency (kHz) | 9000 |
| Mechanical index | 1.30 |
| Radius start (mm) | 12.389 – 15.2 |
| Radius mean step size (mm) | 0.05 – 0.14 |
| Radius min step size (mm) | 0.05 – 0.14 |
| Radius max step size (mm) | 0.05 – 0.14 |
| Radius jitter (mm) | 0.0 |
| Azimuth range (deg) | 110 – 150 |
| Azimuth mean step size (mm) | 0.30 – 0.32 |
| Azimuth min step size (mm) | 0.30 – 0.32 |
| Azimuth max step size (mm) | 0.30 – 0.32 |
| Azimuth jitter (mm) | 0.0 |
| Elevation range (deg) | 118 – 122 |
| Elevation mean step size (mm) | 0.70 – 0.72 |
| Elevation min step size (mm) | 0.70 – 0.72 |
| Elevation max step size (mm) | 0.70 – 0.72 |
| Elevation jitter (mm) | 0.0 |

Table 1c: Settings for shear wave elastography

| **Setting** | **Value** |
| --- | --- |
| Axial voxel size (mm) | 0.09 – 0.10766 |
| Dynamic range (db) | 20 |
| Gain (db) | 14 |
| Power level (%) | 100 |
| Transducer frequency (kHz) | 9000 |
| Mechanical index | 1.4 |
| Radius start (mm) | 12.389 – 15.2 |
| Radius mean step size (mm) | 0.09 – 0.108 |
| Radius min step size (mm) | 0.09 – 0.108 |
| Radius max step size (mm) | 0.09 – 0.108 |
| Radius jitter (mm) | 0.0 |
| Azimuth range (deg) | 110 – 150 |
| Azimuth mean step size (mm) | 0.68 – 0.80 |
| Azimuth min step size (mm) | 0.68 – 0.80 |
| Azimuth max step size (mm) | 0.68 – 0.80 |
| Azimuth jitter (mm) | 0.0 |
| Elevation range (deg) | 119 – 121 |
| Elevation mean step size (mm) | 4.9 – 5.1 |
| Elevation min step size (mm) | 4.9 – 5.1 |
| Elevation max step size (mm) | 4.9 – 5.1 |
| Elevation jitter (mm) | 0.0 |

**References**

1. van den Kroonenberg DL, Jager A, Postema AW, de Bie K, Hagens MJ, Wijkstra H, et al. Development of a computer aided diagnosis system using multiparametric transrectal ultrasound for the localization of clinically significant prostate cancer. The Journal of Urology. 2024;211(5S):e491.

2. van den Kroonenberg DL, Delberghe FT, Jager A, Postema AW, Beerlage HP, Zwart W, et al. Development and Validation of an Algorithm for Segmentation of the Prostate and its Zones from Three-dimensional Transrectal Multiparametric Ultrasound Images. Eur Urol Open Sci. 2025;75:48–54.

3. Jager A, Postema AW, van der Linden H, Nooijen P, Bekers E, Kweldam CF, et al. Reliability of whole mount radical prostatectomy histopathology as the ground truth for artificial intelligence assisted prostate imaging. Virchows Arch. 2023;483(2):197–206.

4. Jager A, Postema AW, Mischi M, Wijkstra H, Beerlage HP, Oddens JR. Clinical Trial Protocol: Developing an Image Classification Algorithm for Prostate Cancer Diagnosis on Three-dimensional Multiparametric Transrectal Ultrasound. Eur Urol Open Sci. 2023;49:32–43.

5. van Leenders G, van der Kwast TH, Grignon DJ, Evans AJ, Kristiansen G, Kweldam CF, et al. The 2019 International Society of Urological Pathology (ISUP) Consensus Conference on Grading of Prostatic Carcinoma. Am J Surg Pathol. 2020;44(8):e87–e99.
